# Use of new targets (D-Mannose receptor, sodium channel voltage dependent) in a new effective, low cost HAART. Validation with the presentation of a clinical case

**DOI:** 10.1186/1742-4690-9-S1-P13

**Published:** 2012-05-25

**Authors:** Adrien Caprani, Guy MK Tran, Laurent Roudiere

**Affiliations:** 1 Alternative Therapies-Basic Science at Association Positifs, Vidauban, France; 2 Clermont-Ferrand University, Hospital Hotel-Dieu, Public Health, Clermont-Ferrand, France; 3 Hopital Pitie Salpetriere, Paris, France

## Background

Neglected data more or less recent, showed that the voltage-dependent sodium channel (Tran MKG) and the Mannose receptor [Bandivdekar AH 14°ISHEID Toulon 2006 (PP 2.14); J Acquir.Immune.Defic.Syndr.2008,Virology.2008] are involved in the transmission of HIV. In particular, the mannose Receptor seem essential for contamination since in a discordant couple, the uninfected male partner does not own this receptor (14° ISHEID, Bandivdekar A.H.). From these facts we have experienced a patient of 68 years, HIV + since 28 years, under active antiretroviral therapy (Epivir, Reyataz, resveratrol) (another presentation at this conference), the following chemotherapy: Epivir (150mg 2daily), resveratrol (500mg 2daily), D-Mannose (1 g 3daily), Omacor (1 g 2daily). Indeed it is known that omega-3fatty acids bind the Na+ channel. Resveratrol has an anti TaT activity (Zhang HS,2009) and a synergy with nucleoside analogues ((HerediaA,2008.)

## Methods

Measurements of viral load , CD4 and CD8 and other usual blood parameters were followed during 9 months, every month.

## Results

Our results show that over a period of 9 months the patient remains undetectable and CD4 count increases significantly from the one of the previous therapy (557+/-43 vs 478+/- 35). Besides, it seems that the CD4/CD8 ratio tended to increase (0.61 vs 0,56 ). Moreover strong activation of the immune system almost always observed among patients HIV+ is standardized at this patient (CD3+/HLADR+ 7%) and the NK strongly increase (24%).

## Conclusion

Our results show the feasibility of HAART including Epivir, resveratrol, D-mannose, and omega3 fatty acids. In addition control of dyslipidemia induced by orthodoxic anti retrovirals should be unnecessary.These results paves the way for clinical trials with effective, low toxicity and low cost agents. Moreover the fact that mutations on the Mannose receptor and sodium channel (cell structures) are unlikely, make the appearance of resistance to such therapies unlikely.

